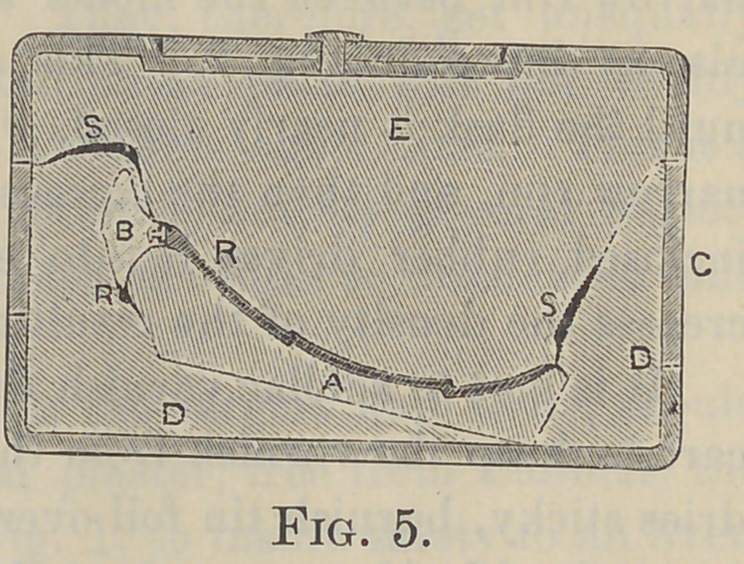# Dental Vulcanite

**Published:** 1896-06

**Authors:** W. Storer How

**Affiliations:** Philadelphia, Pa.


Subject passed.

### Dental Vulcanite.

BY W. STORER HOW, D.D.S., PHILADELPHIA, PA.

Read at the Mississippi Valley Dental Society, April 18th, 1896.

Steel is hardened by heating it to redness and plunging it in water; it is then gradually heated to produce the desired temper-color. The process is so apparently simple that almost any one can do it, yet, long experience and a high degree of skill are required to properly harden and temper steel.

The vulcanizing process is likewise a simple one, but the production of good dental vulcanite is very unusual, because the art in its seeming simplicity is commonly deemed to require but little thought or skill. It is, however, a great mistake to jump to that conclusion, for the making of a fine, strong, solid, odorless vulcanite denture demands careful, intelligent attention to many essential details.

The plaster model should be smooth and hard. The waxing up is to be done neatly and of the right thinness for the vault-part, with a judicious thickness of the ridge and gum-parts which are commonly much too thick. They, therefore, get unequally vulcanized, as the thin parts and thick parts will not evenly harden at the same high heat in the same short time. This is a fundamental fact of the first importance, and its general neglect accounts for the porous, malodorous dentures so often seen and smelt.

A large flask (the Griswold is the largest and best), should be chosen, and with thick-mixed plaster, free from bubbles, the model is set at an angle, as in Fig. 1, to insure safety to an overhanging anterior ridge, making the parting line along the waxed gum border. Shellac varnish the smooth plaster investment and when dried oil that parting surface and pour thick mixed plaster for the mold, jarring the flask to drive the plaster over all the teeth and waxed surfaces. Warm the flask and allow time for the heat to reach and soften the wax, so that the teeth will not be dislodged on separating the flask. Remove the wax and pour boiling water over the model and mold, to melt and wash out the

FIG. 1.

FIG. 2.

remaining wax. Then with alcohol and a camel's hair pencil brush the exposed teeth and pin surfaces to dissolve the wax or water film, which otherwise will prevent adhesion of the vulcanite; and here is disclosed a common cause of loose teeth, the breaking of unsupported teeth and of foul plates. That film also prevents a close fit of the cement stopping of section-joints, and allows the vulcanite to flow between the cement and the sections to make a dirty joint. To provide for the surplus rubber a deep circular groove is cut in the mold-part, leaving a narrow rim between the model and the groove, as 4, in Fig. 1; also as shown in Fig. 2. This allows a free flow of the rubber until the flask is nearly closed, when the flow is checked by the narrow rim, and then the increased pressure distributed over the inclosed rubber drives it into every part of the mold and increases the density of the resulting vulcanite.

It is best to shellac varnish the model and mold (taking great care to keep the varnish from the teeth), and when the shellac dries sticky, burnish tin foil over both model and mold, using a keen-edged knife to cut the foil close around the teeth. This work, nicely done, will, on stripping off the foil after vulcanization, leave a dense, smooth surface, needing very little finishing work. More than that, the surface will be hard and very resistant to the penetration of the oral fluids, or the retention of salivary or alimentary deposits.

A novel and important function of the Griswold flask, Fig. 3, peculiarly adapts it for flasking cases wherein the gum-sections or single teeth have been arranged or ground to fit directly upon the ridge of the model. It is obvious that if the parting line is made between the two parts of the common flask in the usual way, leaving the teeth in the mold-part as shown in Fig. 4, a complete closure of the flask will probably fracture the thin gum of the section (or the thin neck of the plain tooth) by contact with the ridge of the model. If the flask is not quite closed, then the bite will be lengthened or disarranged. But by locking together the base-part B and center C, Fig. 3, the model A and waxed-up teeth B, Fig. 5, can be set in that deep flask so that the investing plaster D shall cover the top of the teeth and make the parting-line at the edge of the center-part C. Then the flask top T may be locked onto the center C, Fig. 5, and plaster poured through the half-round opening on to the shellacked and oiled parting surface to form the mold-part E.

Fig. 3.

Fig. 4.

Fig. 5.

This subsequently serves as a plunger to drive the rubber R, R, into every crevice around the teeth. The grooves for the surplus S, S, should be deeper and nearer the teeth than shown in the sectional view, Fig. 5.

In packing the rubber, there is opportunity for both judgment and skill in cutting suitable pieces, keeping these and the flask-parts warm and clean, and packing the pieces in close contact without much excess of material. The surplus groove should, however, be ample to allow the complete closure of the flask after immersion in very hot water, followed by strong screw-pressure. The closed flask should be securely locked to retain the valuable compressive action for making the vulcanite base dense.

A rubber of well-known purity, strength, color, and vulcanizing heat period is always to be used, but it is a very important fact that the proper heat and time limits are to be varied in accordance with the differing thickness of the several portions of the case in hand; the best results being generally attained by a gradually raised and finally sustained low and long heat. For instance, with a rubber scheduled for one hour at 320° F., a large denture having thin palatal with thick ridge and lip portions will properly require a gradually rising preliminary heat of full forty minutes, and a final period of an hour and a half at 310° F. The surprising excellence of the hard and tough product will well repay the extra expenditure of time and care.

If found necessary (as it seldom should be), to do considerable filing and scraping in finishing such a denture, no holes or stench-traps will be found in that vulcanite, because intelligent provision has been made for the slow and certain interstitial evolution and elimination of the sulphuretted hydrogen which is often confined within the vulcanite skin to form the foul pores and pockets resulting from the commonly crude and quick process.

A first-class automatically regulated vulcanizer is preferable, and the best can be none too good considering the professional work to be done. In any case the vulcanizer should be steam-tight, then water a quarter of an inch deep will be sufficient, and will not boil into the flask to soften the plaster and otherwise cause injury to the vulcanite.

### DISCUSSION.

Dr. Grant Mollyneaux: A series of four articles on this subject, by Dr. Snow, appeared in the *Dental Advertiser* in 1887.

They, without exception, constitute the most thorough treatise on vulcanite I have met with, and with great propriety could be used as text-book literature.

The expansion of vulcanite, incident upon the raising of the temperature to 320° *Fahr.*, is more than seven times that of iron. This expansion is followed by proportionate contraction on cooling, so that a plate made upon a water-soaked cast (already too small) results in a denture entirely too small for the mouth. The plate is subjected to two contractions, one upon cooling, the other, a form peculiar to vulcanite itself. The plate exhibits a convex surface when taken from the cast, the result being that contact first obtains on the hard palate, producing rocking. In experiments that were made, vulcanizing on a plain surface of plaster, this convexity of form in the vulcanized piece of rubber was shown by its lifting away at the sides from the square of plaster and remaining in contact with it in the middle of the square. This refers to the sheet of rubber placed on the top of the plaster square. Another sheet, vulcanized on the bottom of the block, showed the same result, the plaster being forced down into the center of the sheet, constituting a depression in it, while the edges turned up as in the case of the sheet upon the top of the block. To obviate warpage, in making repairs, I place the flask in the vulcanizer with the teeth *down*, next to flame—the philosophy of this is readily apparent, the pressure from expansion being upward and against the sides of the cast, and should a space result it will be at the center of the vault, and is desirable because it obviates the rocking above explained.

## Figures and Tables

**Fig. 1. f1:**
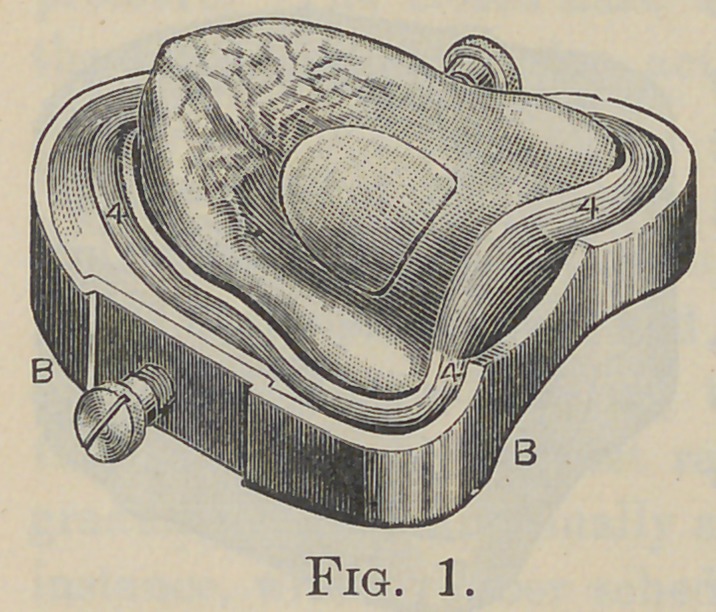


**Fig. 2. f2:**
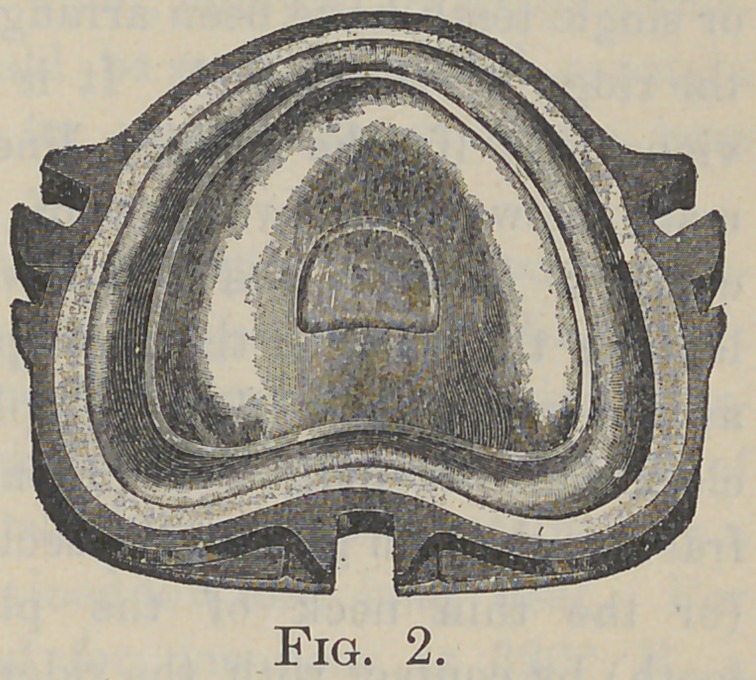


**Fig. 3. f3:**
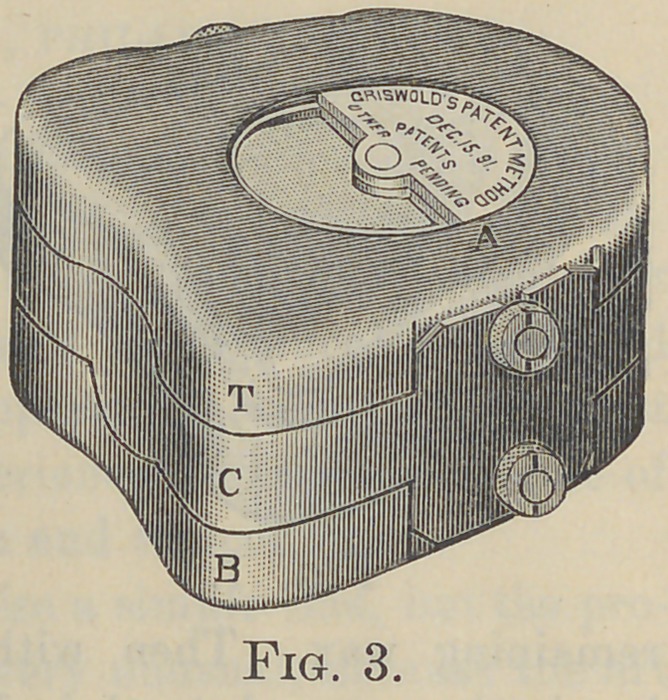


**Fig. 4. f4:**
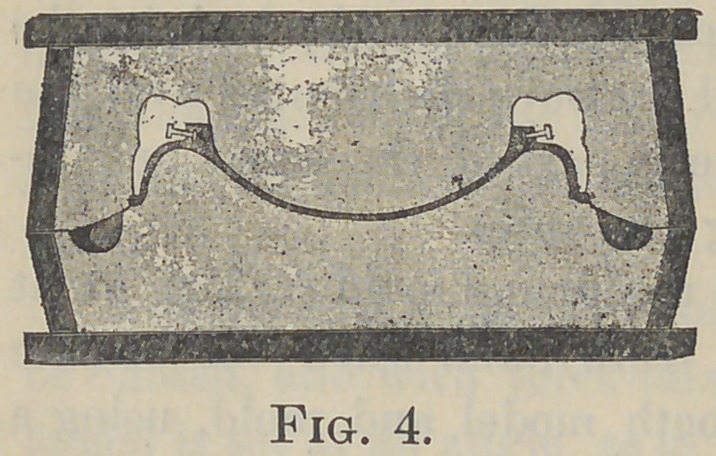


**Fig. 5. f5:**